# Supplementary material for: Does higher sampling rate (multiband + SENSE) improve group statistics - An example from social neuroscience block design at 3T
Source: Neuroimage. 2020 Jun;213:116731. doi: 10.1016/j.neuroimage.2020.116731 (PMC7181191; doi:10.1016/j.neuroimage.2020.116731)
Supplement: Multimedia component 1 [file mmc1.docx]

**Supplementary Materials**

**Supplementary table 1** List of the hand actions used in action observation. The actor’s right hand always entered the screen and initiated the goal-directed action from the left side of the screen. Note that control movies showed the same objects used for action observation but the actor’s hand moved close to but without interacting with the object.

| **N◦** | **Recorded actions description** | **Movie**  **Duration** |
| --- | --- | --- |
| 1 | Stirring coffee with a spoon. | 2s |
| 2 | Putting a cube of sugar in a cup of coffee. | 2s |
| 3 | Closing a box with a key. | 2s |
| 4 | Lighting a candle with a lighter. | 2s |
| 5 | Putting a flower in a vase | 2s |
| 6 | Putting a battery in a remote control. | 2s |
| 7 | Putting a CD in a CDs stack. | 3s |
| 8 | Hammering a nail. | 3s |
| 9 | Putting whipped cream on strawberries. | 3s |
| 10 | Cutting a deck of cards. | 2s |
| 11 | Placing jewelry in a box. | 2s |
| 12 | Crumpling a paper sheet. | 2s |
| 13 | Closing a box of chewing gums. | 2s |
| 14 | Putting a pin on a foam base. | 2s |
| 15 | Taking hand cream from a tin. | 2s |
| 16 | Taking some tape and placing it on a box. | 3s |
| 17 | Pouring wine in a glass | 3s |
| 18 | Watering a plant. | 3s |
| 19 | Stirring eggs. | 2s |
| 20 | Closing a water bottle. | 2s |
| 21 | Flipping through a block note. | 2s |
| 22 | Taking an olive from a jar. | 2s |
| 23 | Putting a candle in a candleholder. | 2s |
| 24 | Closing a folder. | 2s |
| 25 | Cracking walnuts. | 3s |
| 26 | Placing a wine bottle in a box. | 3s |
| 27 | Opening a suitcase. | 3s |
| 28 | Spreading jam on a piece of bread. | 2s |
| 29 | Cutting a ribbon on a package. | 2s |
| 30 | Stirring soup with a spoon. | 2s |
| 31 | Putting business cards in a box. | 2s |
| 32 | Putting a hair clip in a purse. | 2s |
| 33 | Disconnecting headphones from an MP3 player. | 2s |
| 34 | Breaking an egg on the edge of a bowl. | 3s |
| 35 | Stirring a painting brush in a cup of water. | 3s |
| 36 | Taking a walnut with chopsticks and placing it in a box. | 3s |

In Supplementary Analysis 1 – 4 we present additional analysis of factors which may impact the effect of the MB acceleration on the random effect analysis.

**Supplementary Analysis 1: G-factor**

To get an estimate of the aliasing noise that results from sub-optimal separation of the simultaneously acquired voxels, g-factor maps were inspected. G-factor values closer to 1 are considered “clean” and higher values in the voxels represent more noise. Supplementary figure 1 shows g-factor maps from a representative subject for ${MB1S2}_{2.7iso}^{2.45}$, ${MB2S2}_{2.7iso}^{1.22}$, ${MB4S1.5}_{2.7iso}^{0.70}$. The histogram of these maps is shown in supplementary figure 2. As expected, the aliasing noise increases with higher MB factors. While the noise amplification in ${MB2S2}_{2.7iso}^{1.22}$ is restricted to a small region (hot spots in the posterior part of the images, row 2), the sequences with ${MB4S1.5}_{2.7iso}^{0.70}$ show more widespread g-factors above 1.2 (yellow in top row). Row 3 in supplementary figure 1 shows the g-factor values for ${MB1S2}_{2.7iso}^{2.45}$ and the values fall much closer to 1 - as expected, since there are no simultaneously acquired voxels. Table 3 presents the average g-factor mode and the 99 percentile values over all voxels and subjects. As in the single subject maps, at the group level we can see an increase in g-factor mode from MB1 to MB4. To understand the effect of g-factor penalty on tSNR, we calculated the relative expected tSNR (tSNR_rel_). Practically, tSNR_rel_ was computed per voxel relative to our reference sequence with given TR_ref_=2.45 and SENSE factor S_ref_=2 without MB implementation using the following equation.

${tSNR}_{rel,i}=\frac{1}{g_{i,MB}\sqrt{\frac{TR}{{TR}_{ref}}\cdot\frac{S}{S_{ref}}}}$

Here, g_i,MB_ is the g-factor value in voxel i, for a certain sequence. Supplementary table 2 presents the tSNR_rel_ mode and the 99 percentile values over all subjects. We find that despite higher g values, the predicted tSNR_rel_ increases in sequences with higher MB factor. While the higher g-factor values speak against the use of higher MB factors, in theory, reduction in TR leading to more data points per unit time, would ultimately result in an 87% (√(2.45/0.70)-1) increase in effective SNR going from MB1 to MB4. This can be seen to be true in tSNR_rel_ values. Thus, if we base our conclusion on these matrices, scanning at higher MB-factors is preferable.


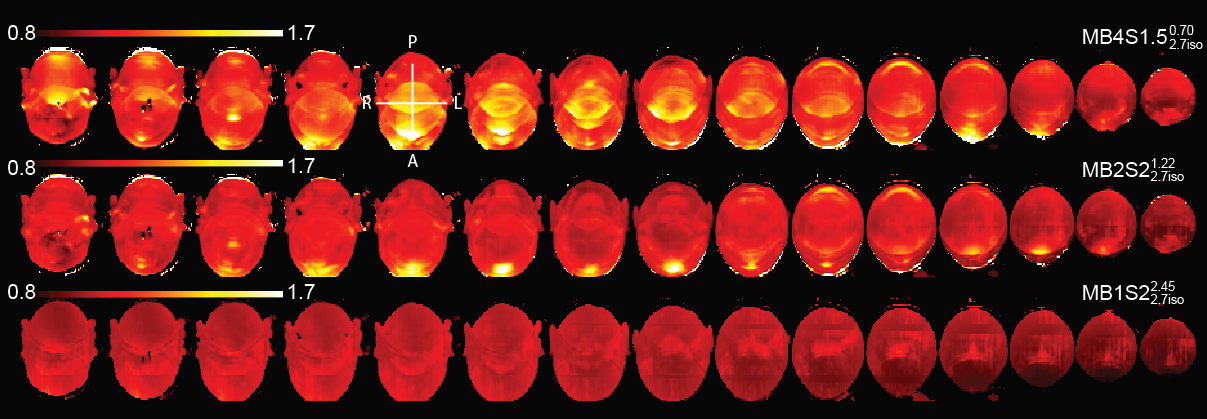


**Supplementary Figure 1.** Raw G-factor images from a representative subject for sequences ${MB1S2}_{2.7iso}^{2.45}$, ${MB2S2}_{2.7iso}^{1.22}$ and ${MB4S1.5}_{2.7iso}^{0.70}$. G-factor values close to 1 (red) represent minimal aliasing noise while increase in this value (yellow) indicate suboptimal separation of simultaneously acquired voxels. High values are rare at TR 2.45s, restricted in TR 1.22s but widespread in TR 0.70s.


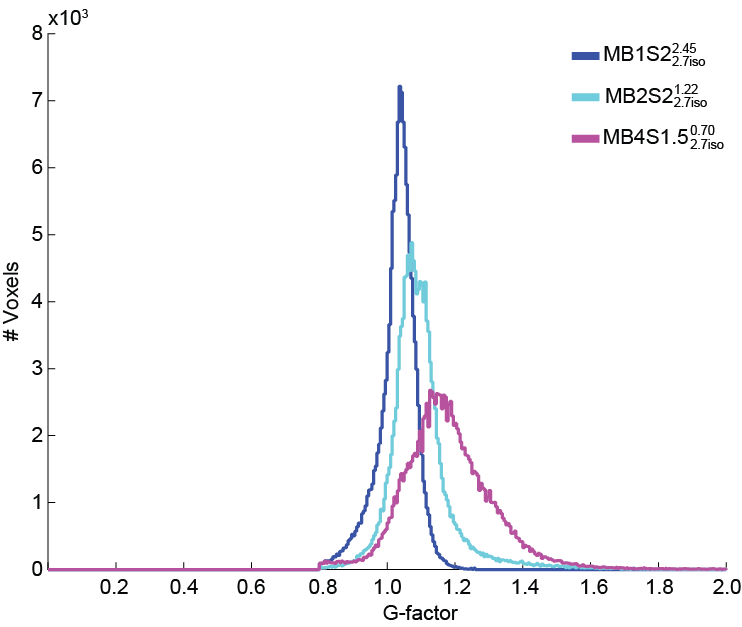


**Supplementary Figure 2.** Histogram of images in Supplementary figure 1 from a representative subject confirm that the g-factor values increase when higher MB factor is implemented.

**Supplementary table 2.** G-factor and relative tSNR (predicted)

| TR | | ${MB1S2}_{2.7iso}^{2.45}$ | ${MB2S2}_{2.7iso}^{1.22}$ | ${MB4S1.5}_{2.7iso}^{0.70}$ |
| --- | --- | --- | --- | --- |
| metric | statistic | Mean ± SD over subjects | | |
| G-factor | mode | 1.04 ± 0.01 | 1.09 ± 0.02 | 1.17 ± 0.03 |
|  | 99 percentile | 1.14 ± 0.02 | 1.52 ± 0.07 | 1.65 ± 0.05 |
| tSNR_rel_ | mode | 0.96 ± 0.01 | 1.30 ± 0.02 | 1.85 ± 0.04 |
|  | 99 percentile | 0.88 ± 0.02 | 0.93 ± 0.04 | 1.31 ± 0.04 |

**Supplementary Analysis 2: Effective Temporal Signal to Noise Ratio (effective tSNR)**

While the tSNR_rel_ gives a theoretical prediction of the tSNR that can be expected based on data from the initial calibration, we next used the fMRI data to get the actual effective voxel-wise tSNR as described in Todd et al. (2016). We divided the mean signal over time after removal of the task based activity (i.e. m; the constant term from the first level GLM fit, see section 2.6) by the standard deviation (σ) over time of the residual signal after the GLM fit (section 2.6). We then scaled this by a factor that corrects for the different number of volumes acquired with different sequences and accounts for the autocorrelations in the data using the following equation.

$tSNR=\frac{m}{\sigma}.\sqrt{\frac{N}{k}}$

Here, N = number of volumes, k = c^T^’ (X^T^’X) c, where, X = whitened and high pass filtered design matrix and contrast (c) = [0 0 0 0 0 0 0 0 0 0 0 0 0 0 0 0 0 0 1] (i.e. only considering the global factor that captures the average activity after task removal).

The tSNR maps per subject were then masked with a grey matter mask and then averaged across subjects, separately for each acquisition sequence. Supplementary figure 3 presents the average tSNR maps. Visual comparison of the maps shows that the tSNR values are higher with higher acceleration. A within-subject ANOVA with the mean tSNR value in the gray-matter per subject showed a main effect of TR (*F* (4, 92) = 8.61, *p*<0.001) (Supplementary figure 4). Keeping voxel size constant, tSNR values increase with higher MB (${MB1S2}_{2.7iso}^{2.45}$ < ${MB2S2}_{2.7iso}^{1.22}$ < ${MB4S1.5}_{2.7iso}^{0.70}$ < ${MB4S2}_{2.7iso}^{0.63}$). Comparing these values against ${MB1S2}_{3x3x3.3}^{2.00}$ sequence shows that MB2 can compensate for a 50% reduction in voxel volume, and that MB4 provides highest tSNR.


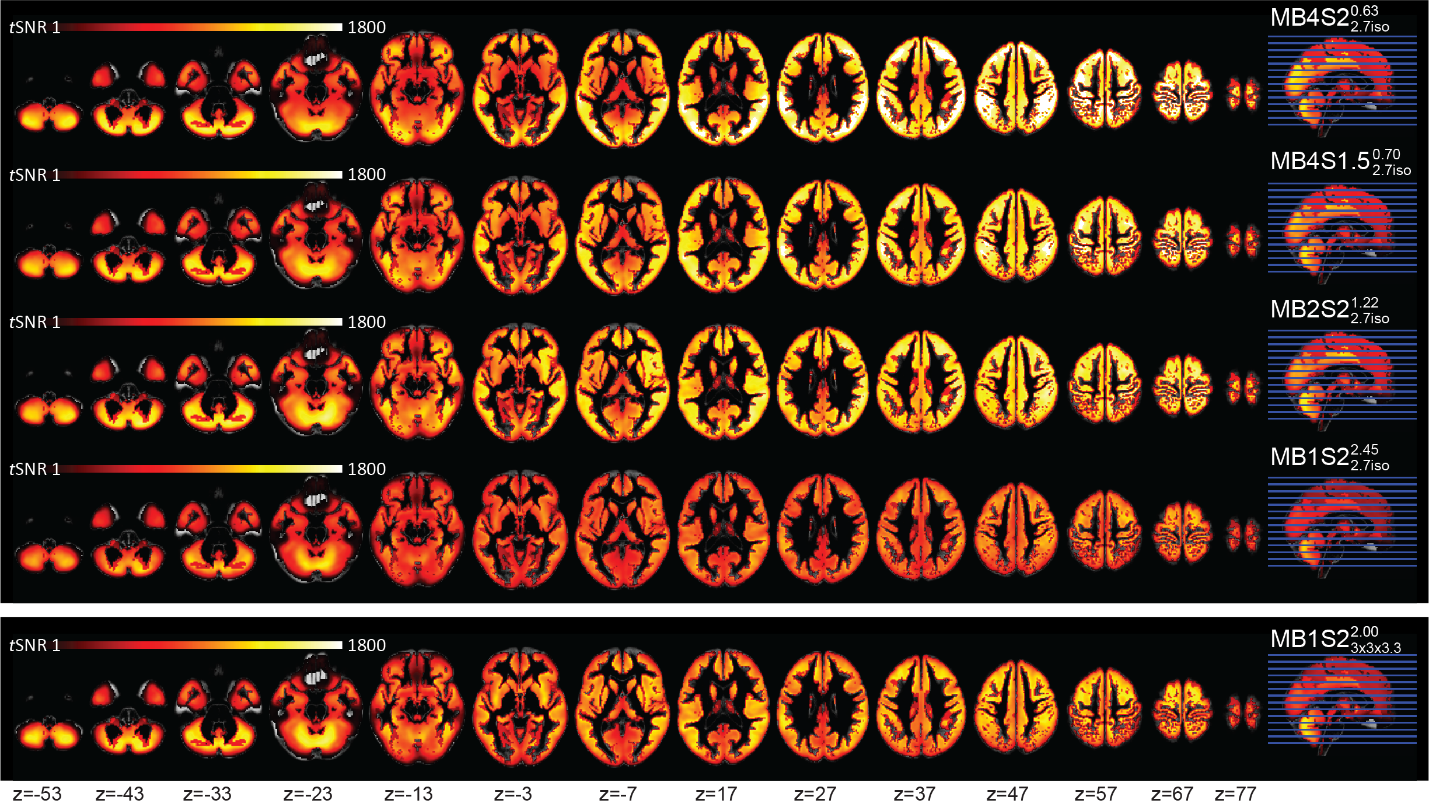


**Supplementary Figure 3.** Average TSNR maps (within grey matter mask) as a function of TR. In general, tSNR values appear to be higher for sequences with MB implementation.


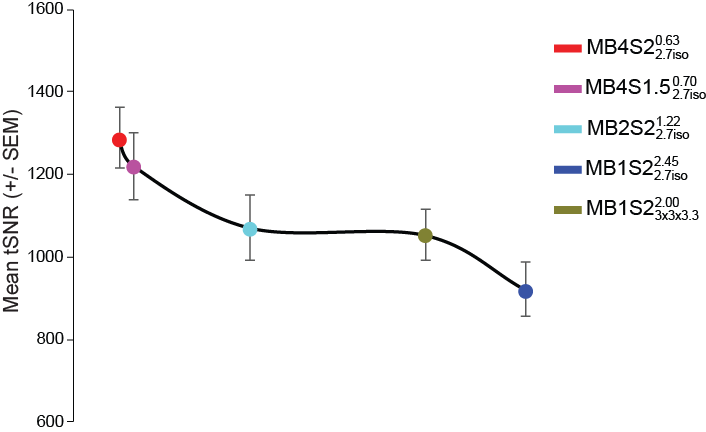


**Supplementary Figure 4.** Plot of the mean tSNR values (+/- sem over the subjects) within the grey matter mask confirms that values are higher with higher MB. ${MB4S2}_{2.7iso}^{0.63}$ shows highest tSNR values.

**Supplementary Analysis 3: Raw Temporal Signal to Noise Ratio**

Supplementary figure 5 presents the subject average raw tSNR maps separately for each sequence. Briefly, the temporal mean and the temporal standard deviation of the preprocessed functional data (i.e. slice-time corrected--realigned—normalized--smoothed) were calculated for each subject. The Raw tSNR was calculated using the following equation. We then calculated the average tSNR maps across subjects.

$$\boldsymbol{Raw tSNR=}\frac{\boldsymbol{temporal mean per voxel}}{\boldsymbol{temporal standard deviation per voxel}}$$

**
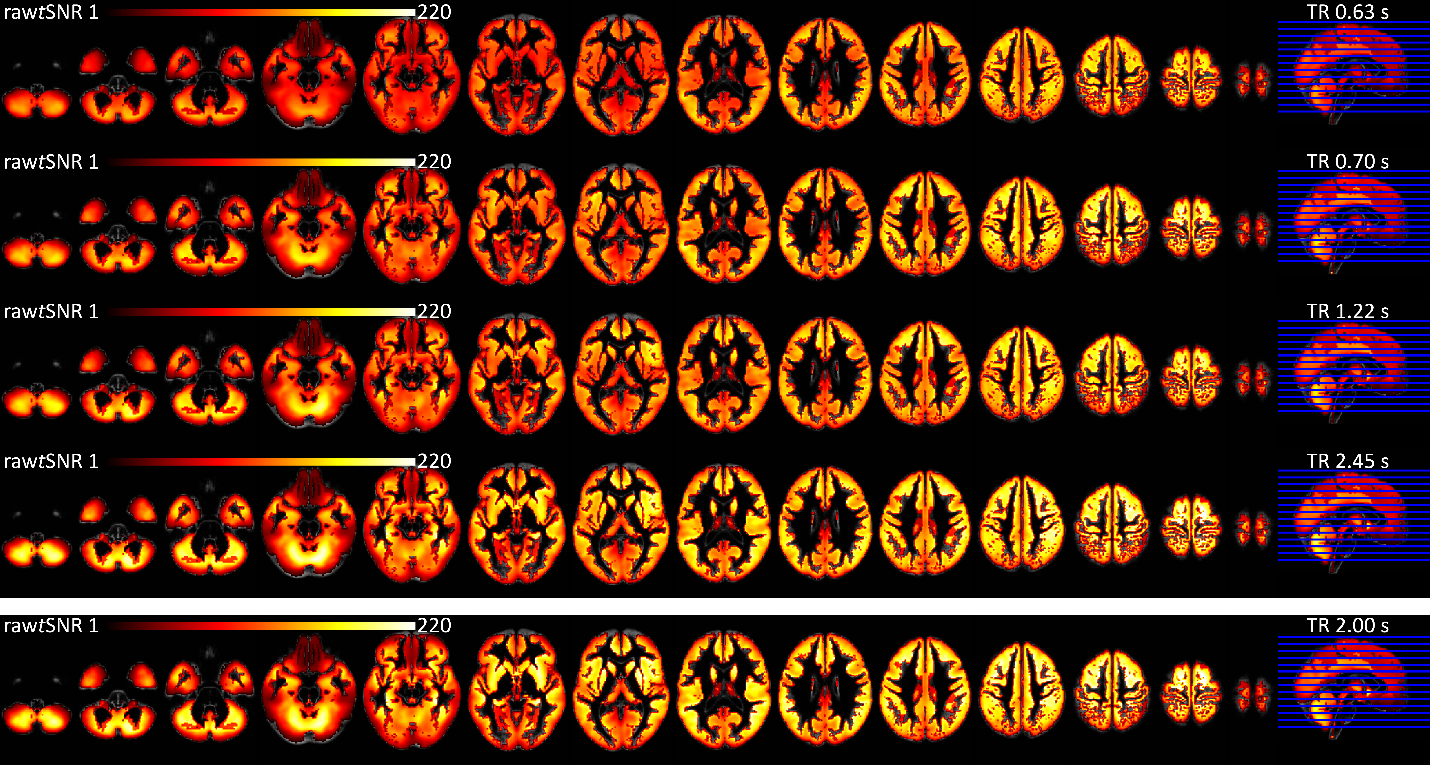
**

**Supplementary Figure 5.** Average raw TSNR maps (within grey matter mask) as a function of TR. In general, tSNR values appear to be lower for sequences with MB implementation.

**Supplementary Analysis 4: Contrast to Noise Ratio (CNR)**

Supplementary figure 6 shows the mean functional EPI as a function of sequence from one representative participant. As can be seen, the white/gray matter contrast is lower for sequences with short er TRs. For each subject, to quantify this white/gray matter contrast loss, CNR was calculated on the mean functional image (created during the realigning procedure). The mean functional image was separated in white and gray compartments using individual gray and white matter masks obtained from the segmentation of the co-registered T1 image (thresholded to include values > 0.8 of estimated probability). We then calculated the average and standard deviation separately for the gray and white matter compartments. The contrast to noise ratio was then calculated by dividing the signal (i.e. the difference in mean between the white and gray matter compartments) by the noise (i.e. the standard deviation of the union of the demeaned gray and white matter voxels) as in the following equation.

$CNR=\frac{\left( Mean gray matter-Mean white matter \right)}{STD \left( Gray matter-Mean gray matter+White matter-Mean white matter \right)}$

A within-subject ANOVA to test the difference between the CNR values of the five acquisition schemes revealed a highly significant main effect of MB (*F* (4, 84) =512.53, *p* < 0.001) on CNR due to a decrease in CNR with higher TR (Supplementary figure 7). While the decrease was mostly linear with decreasing TR, the CNR values for ${MB4S2}_{2.7iso}^{0.63}$ were slightly better than ${MB4S1.5}_{2.7iso}^{0.70}$, suggesting that in plane acceleration of SENSE 2 may afford slightly better grey-white contrast than SENSE 1.5.


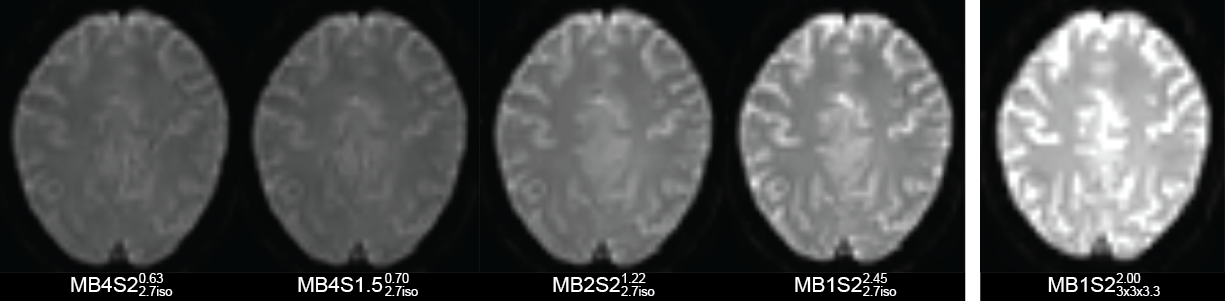


**Supplementary figure 6.** Mean EPIs from a representative subject estimated during the realignment procedure. The images are not normalized and they correspond to z=31. The grey white contrast seems to decrease for short er TRs and artifacts become evident for MB>2.


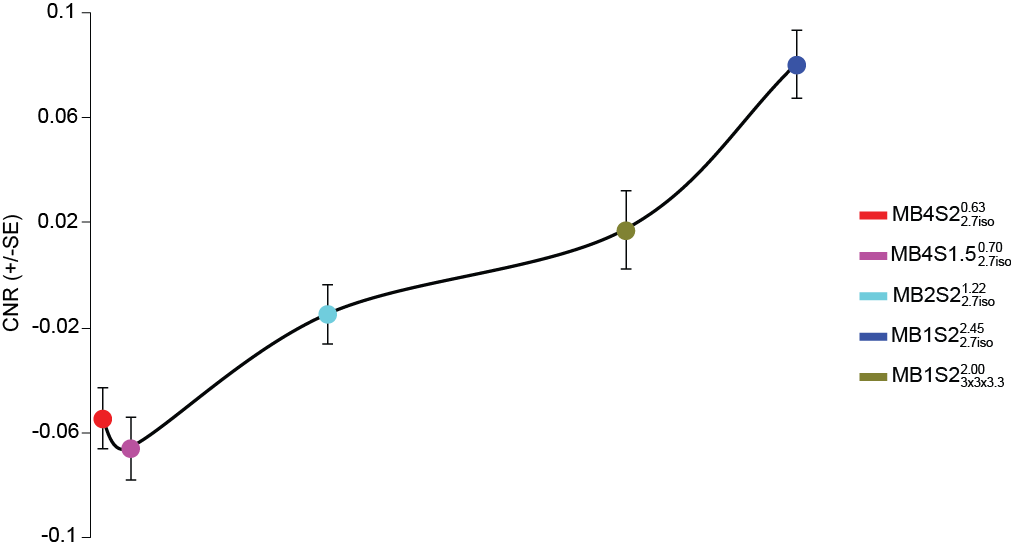


**Supplementary figure 7.** CNR of the white/gray matter shows a significant main effect of MB (*F*(4, 84) = 512.5, *p*< 0.001) confirming a decrease in contrast values as the TR becomes shorter.

**Supplementary Analysis 5: Second-level t-test with 22 subjects**

Second level t-tests were performed with 22 subjects for each sequence, making the sample size equal to that with MB2 acceleration. Histograms of the t-values from these t-tests are presented in supplementary figure 8. It is apparent that that the overall results and conclusions do not chance when a sample of 22 vs 23 is used. See figure 2 D for comparison.


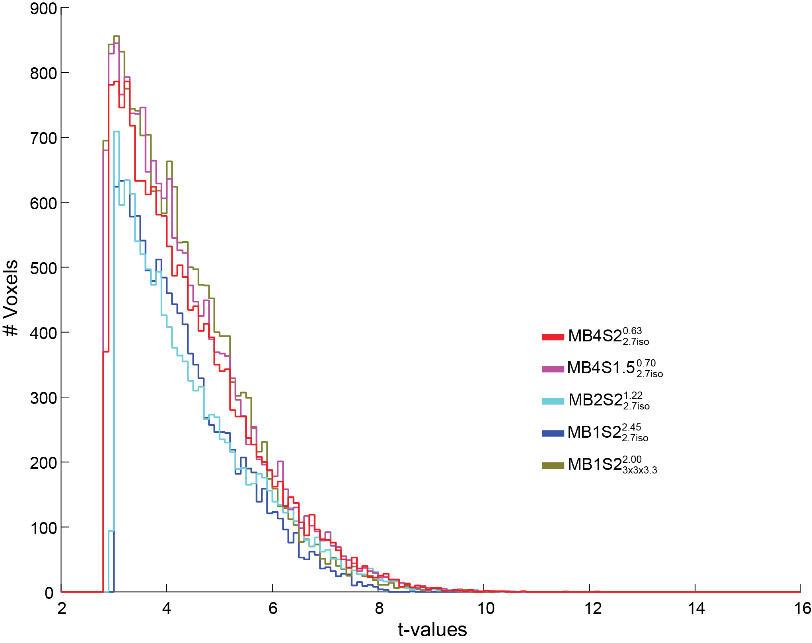


**Supplementary figure 8.** If we use 22 subjects for each TR, making the sample size same in all sequences, the overall t-values and their distribution between different sequences does not change.

**Supplementary Analysis 6: Modelling the haemodynamic response with canonical HRF and the temporal and dispersion derivatives**

To see if alternative models of the haemodynamic response affect our interpretation of the benefits of MB acceleration, we convolved the predictors with the HRF and the temporal and dispersion derivatives that allow us to capture differences in the latency and the duration of the peak response, respectively. Briefly, at the level 1 modeling, we used the option to convolve the temporal and dispersion derivatives. This resulted in three predictors for the CA condition (1^st^ convolved with canonical HRF, 2nd with temporal derivative and the third with dispersion derivative). Similarly, three predictors were used for the CC condition. All other settings were the same as described in section 2.6. The model was estimated and then we calculated the amplitude estimate that is a function of canonical HRF and the derivatives as described in Calhoun et al., 2004, using the following equation.

$$sign\left( \beta1-\beta4 \right).*\left( \sqrt{\left( \left( \beta1-\beta4 \right)^{2}+ \left( \beta2-\beta5 \right)^{2}+\left( \beta3-\beta6 \right)^{2} \right)} \right)$$

Here, β1 to β3 are the beta maps for the canonical HRF, temporal derivative and the dispersion derivative for the CA predictors and β4 to β6 are the same for the CC predictor. The resulting maps were the CA-CC contrast maps and included the amplitude estimate that is a function of both the non-derivative and the derivative terms of the model. Group level t-test were performed as described in section 2.6.

Supplementary figure 9 presents the maps, separately for each sequence and the histogram of the t-values. As can be see when compared to the maps in figure 2A, the network recruited does not change extensively when the derivatives are modelled. Similarly, looking at the histogram shows that sequences with MB4 still outperforms sequences with lower MB in the same way as shown in figure 2D.


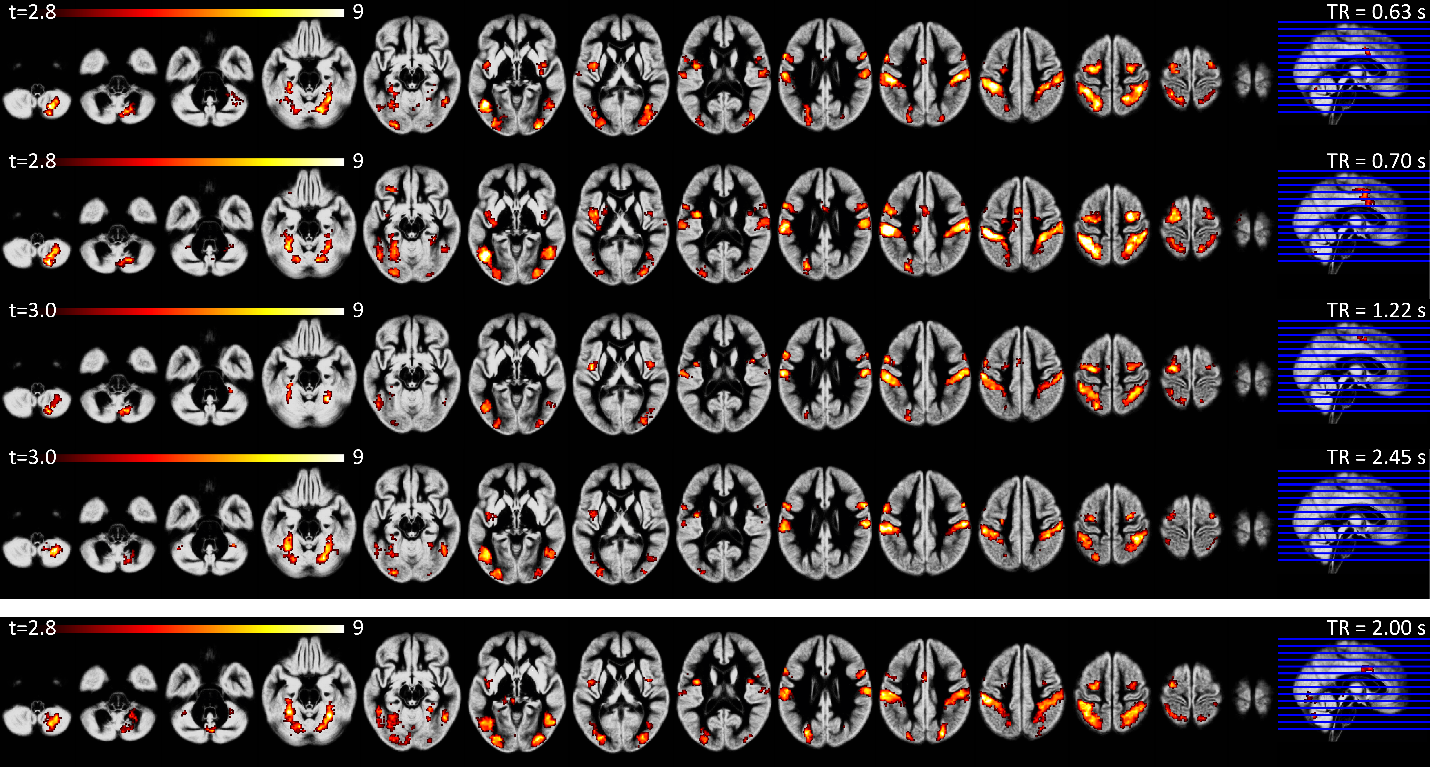


**
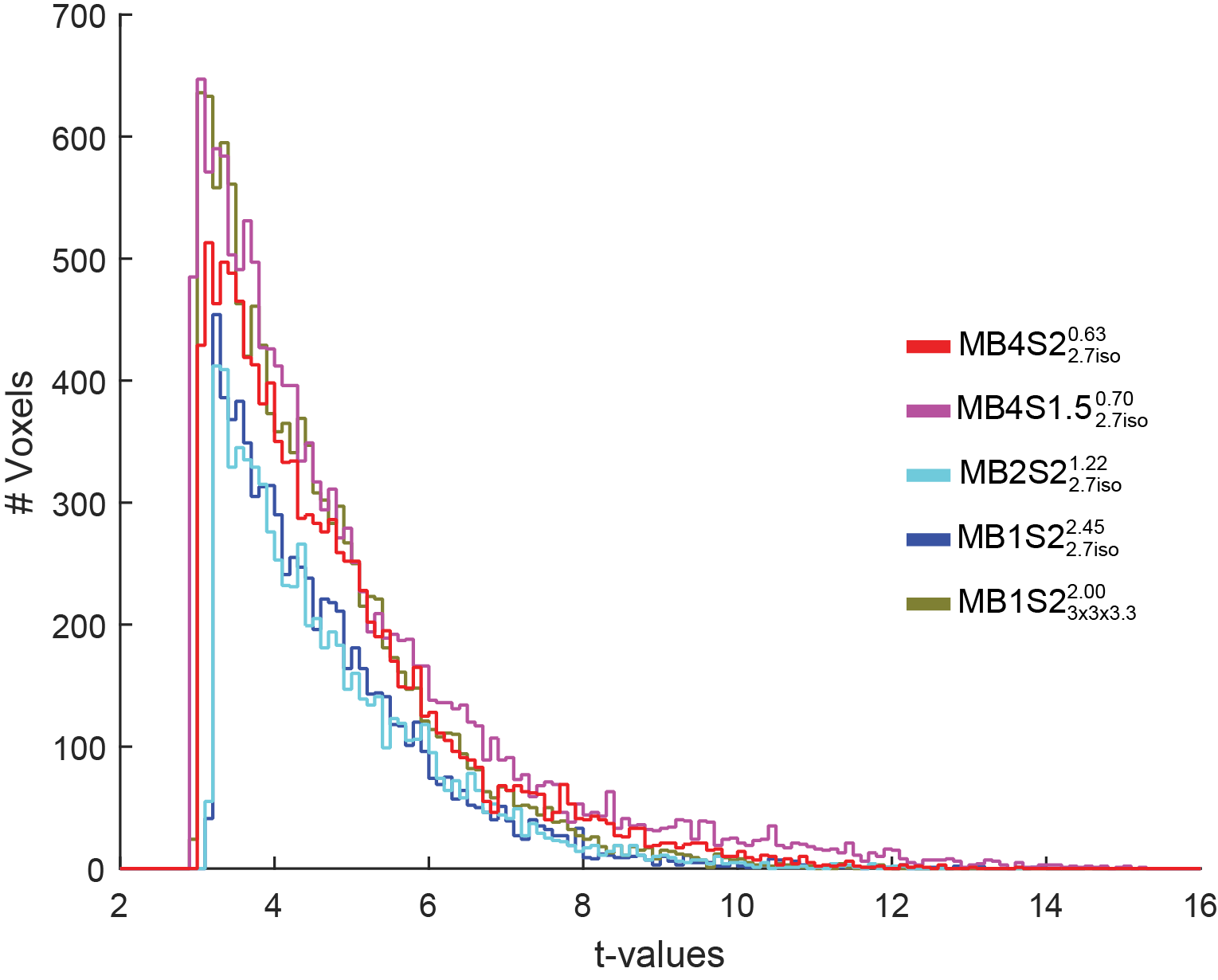
**

**Supplementary figure 9.** Maps and histogram showing the network and t-value distribution when canonical HRF and the temporal and dispersion derivatives are modelled (N=22).

**Supplementary Analysis 7: Results of group level ANOVA presented at p_uncorr_ < 0.001**

Since FDR correction leads to different t-thresholds for each sequence, we also present the uncorrected results in supplementary figure 10.


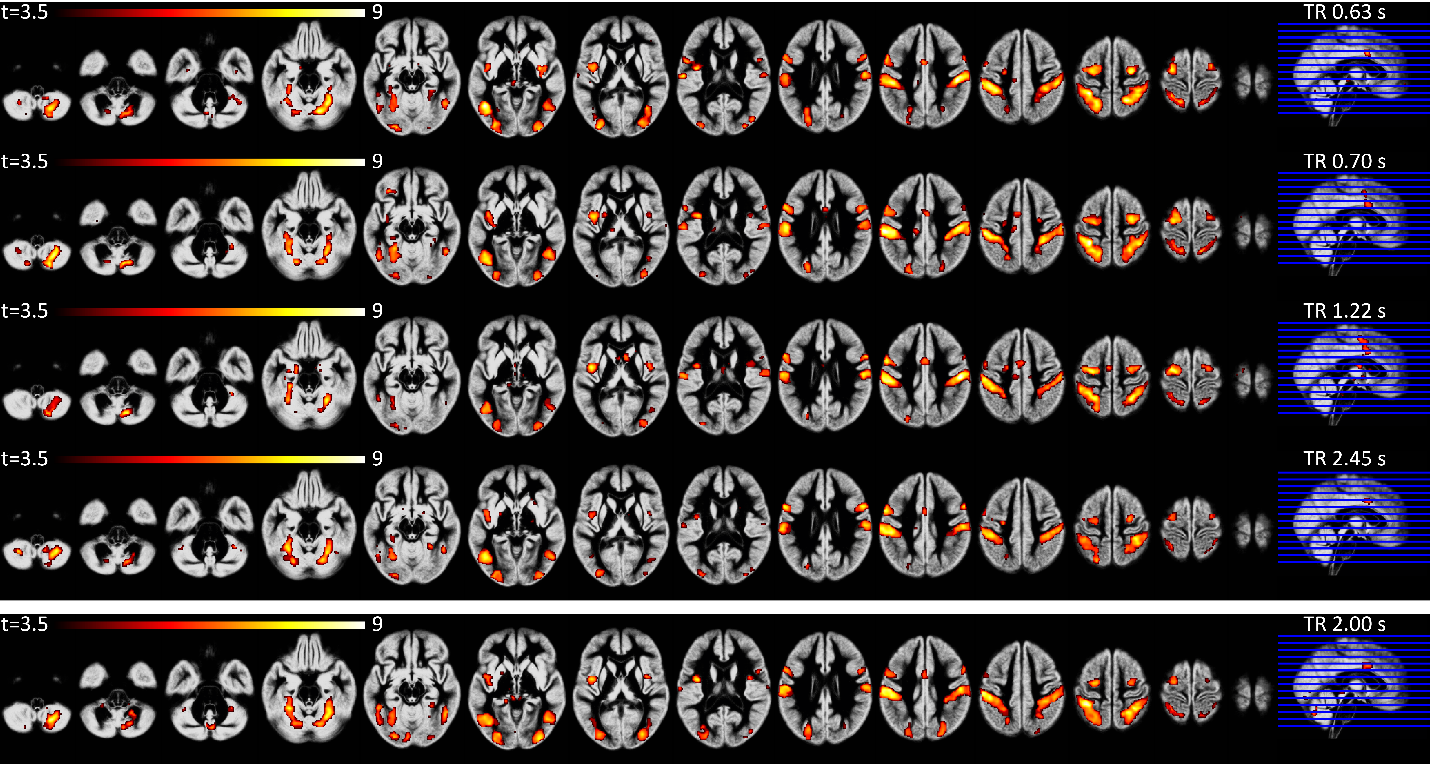


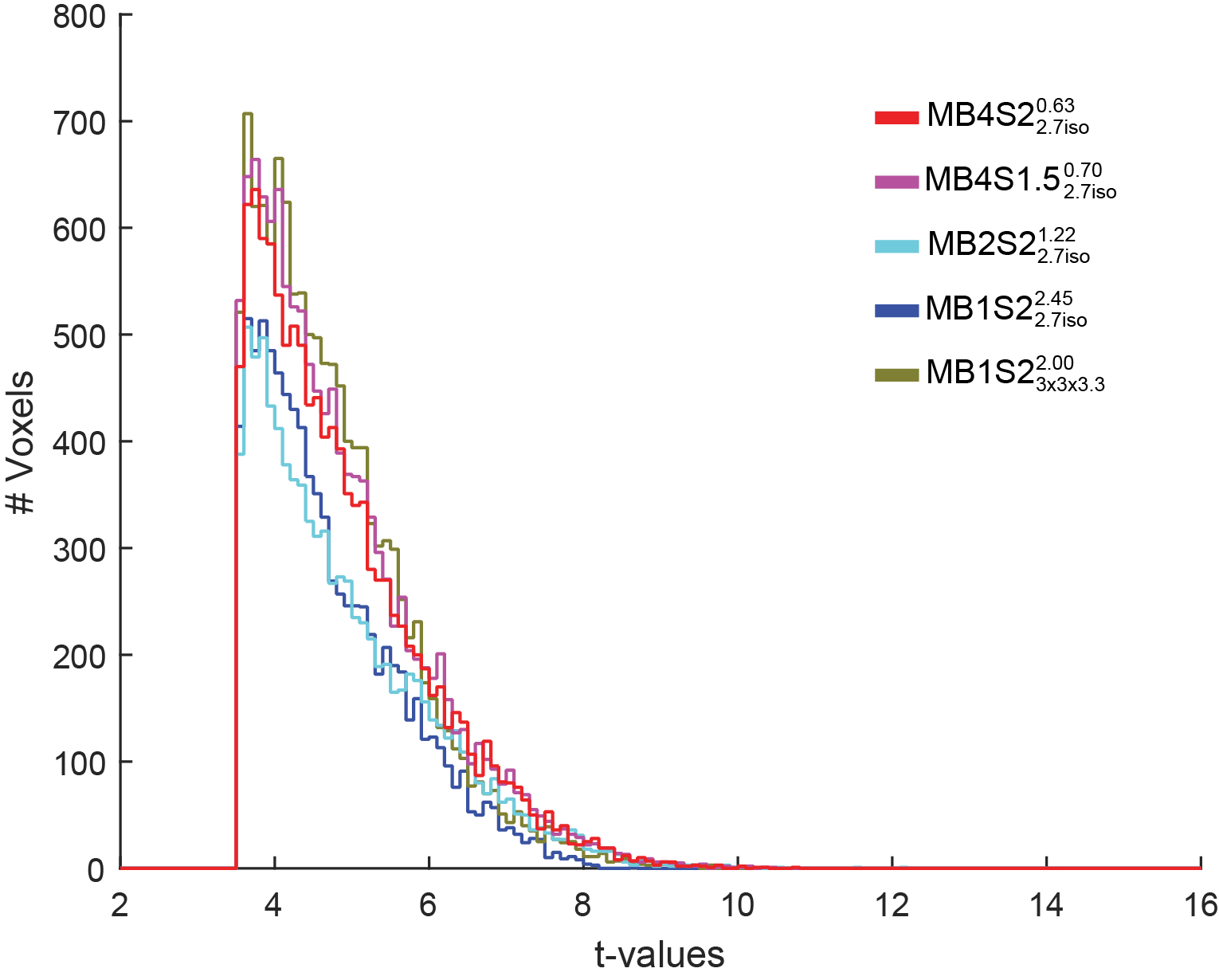


**Supplementary figure 10.** Maps and histogram showing the network and t-value distribution when no correction is applied (N=22).
